# An Update of Recent Use of *Aegilops* Species in Wheat Breeding

**DOI:** 10.3389/fpls.2019.00585

**Published:** 2019-05-09

**Authors:** Masahiro Kishii

**Affiliations:** Global Wheat Program, International Maize and Wheat Improvement Center (CIMMYT), Texcoco, Mexico

**Keywords:** *Aegilops*, introgression, translocation, wheat breeding, stress tolerance, wild species, genetic resources

## Abstract

*Aegilops* species have significantly contributed to wheat breeding despite the difficulties involved in the handling of wild species, such as crossability and incompatibility. A number of biotic resistance genes have been identified and incorporated into wheat varieties from *Aegilops* species, and this genus is also contributing toward improvement of complex traits such as yield and abiotic tolerance for drought and heat. The D genome diploid species of *Aegilops tauschii* has been utilized most often in wheat breeding programs. Other *Aegilops* species are more difficult to utilize in the breeding because of lower meiotic recombination frequencies; generally they can be utilized only after extensive and time-consuming procedures in the form of translocation/introgression lines. After the emergence of Ug99 stem rust and wheat blast threats, *Aegilops* species gathered more attention as a form of new resistance sources. This article aims to update recent progress on *Aegilops* species, as well as to cover new topics around their use in wheat breeding.

## Introduction

According to the latest revision of *Aegilops* L. taxonomy, (van Slageren system) which is used by most researchers (including this article), *Aegilops* consists of 23 species, having the D, S, U, C, N, and M genomes (van Slageren, 1994). Since the taxonomy has frequently changed (Kihara, 1954; Hammer, 1980; Witcombe, 1983; Kimber and Sears, 1987; van Slageren, 1994) this has led to some confusion about species names, and so a list of *Aegilops* species is provided below (Table 1). The biggest change from the previous taxonomy system is that *Ae. mutica* Boiss. has been removed and assigned a new species name: *Ambylopyrum muticum* (Boiss.) Eig. In the future, it will also be possible to make further modifications to reflect molecular findings (Edet et al., 2018).

**Table 1:** Taxonomy and genomic constitution of *Aegilops* species.

Genome^∗^	Taxonomy system			
	
	van Slageren, 1994	Witcombe, 1983	Hammer, 1980	Kimber and Sears, 1987
D	*Ae. tauschii*	*Ae. squarrosa*	✓	*Triticum tauschii*
S	*Ae. speltoides*	✓	✓	*T. speltoides*
”	”	*Ae. ligustica*	✓	*T. speltoides*
S^b^	*Ae. bicornis*	✓	✓	*T. bicorne*
S^l^	*Ae. longissima*	✓	✓	*T. longissimum*
S^sh^	*Ae. sharonensis*	✓	*Ae. longissima*	*T. sharonense*
S^s^	*Ae. searsii*	✓	✓	*T. searsii*
C	*Ae. caudata*	✓	*Ae. markgrafii*	*T. dichasians*
M	*Ae. comosa*	✓	✓	*T. comosum*
N	*Ae. uniaristata*	✓	✓	*T. uniaristatum*
U	*Ae. umbellulata*	✓	✓	*T. umbellulatum*
CD	*Ae. cylindrica*	✓	*Ae. cylindrica*	*T. cylindricum*
DN	*Ae. ventricosa*	✓	✓	*T. ventricosum*
DM	*Ae. crassa*	✓	✓	*T. crassum*
DDM	”	✓	✓	*T. crassum*
DMS	*Ae. vavilovii*	✓	*Ae. crassa*	*T. syriacum*
DMU	*Ae. juvenalis*	✓	✓	*T. juvenale*
US	*Ae. peregrina*	✓	✓	*T. peregrinum*
US	*Ae. kotschyi*	✓	✓	*T. kotschyi*
UC	*Ae. triuncialis*	✓	✓	*T. triunciale*
UM	*Ae. biuncialis*	*Ae. lorentii*	*Ae. lorentii*	*T. macrochaetum*
UM	*Ae. columnaris*	✓	✓	*T. columnare*
UM	*Ae. geniculata*	*Ae. ovata*	✓	*T. ovatum*
UM	*Ae. neglecta*	*Ae. triaristata*	✓	*T. neglectum*
UMN	”	*Ae. triaristata*	✓	*T. rectum*
T	*Amblyopyrum mutica*	*Ae. mutica*	*Ae. mutica*	*T. tripsacoides*
	–	–	*Ae. turcomanica*	–


*^∗^Genome symbols follow to Waines and Barnhart (1992).”, same as the above; ✓, same species name to van Slageren (1994).*

One of the most important aspects of *Aegilops* is that it is closely related to bread wheat *Triticum aestivum* L. (AABBDD), which is one of the most important calorie sources for human nutrition. The D genome originated from the diploid species of *Aegilops tauschii* Coss. ( = *Ae. squarrosa* L.) (Kihara, 1946; McFadden and Sears, 1946), and the B genome was derived from a closely related species to *Ae. speltoides* Tausch (Riley et al., 1958; Sasanuma et al., 1996; Petersen et al., 2006; Kilian et al., 2007; Zhang W. et al., 2018) which has the S genome. *Aegilops* species are distributed from Europe to western China in a species-specific manner (van Slageren, 1994), adapted to many different climatic zones including drought/heat environments, different disease hot spots and nutrient-poor areas. It has been reported that *Aegilops* possesses useful traits for wheat breeding (For review to see; Kilian et al., 2011) including drought tolerance (Damania et al., 1992; Waines et al., 1993; Rekika et al., 1998; Monneveux et al., 2000; Farooq and Azam, 2001), heat tolerance (Waines, 1994), salinity (Colmer et al., 2006), aluminum toxicity tolerance (Miller et al., 1995) and resistance to several pests and diseases such as rust (Mihova, 1988; Anikster et al., 2005; Liu et al., 2010; Rouse et al., 2011; Vikas et al., 2014; Huang S. et al., 2018; Olivera et al., 2018), powdery mildew (Lutz et al., 1994; Buloichik et al., 2008), Hessian fly (El Bouhssini et al., 2008), cereal aphid (Holubec and Havlıckova, 1994) and barley yellow dwarf virus (BYDV) (Makkouk et al., 1994). In addition, the species can adapt to low phosphorous environments (Liu et al., 2015) and can contribute to higher iron and zinc content in wheat grain (Rawat et al., 2009).

In order to effectively exploit these useful traits in wheat, it is necessary to overcome extra difficulties with the introgression process, including a hybridization barriers, incompatibilities/hybrid abnormalities, sterility of F_1_s and, reduced meiotic chromosome pairings. Despite these obstacles, many *Aegilops* genes have been transferred to wheat and have been heavily utilized over the last 60 years (For review to see; Schneider et al., 2008; Kilian et al., 2011). *Aegilops* is also contributing to abate two recent threats to the global wheat production: Ug99 stem rust race derivatives and wheat blast (*Magnaporthe oryzae Triticum*). When Ug99 (original pathotype TTKSK) appeared in the early 2000s (Pretorius et al., 2000), more than 80% of wheat varieties did not have resistance against the race (Pretorius et al., 2000) and as such, wheat breeders sought resistance traits in *Aegilops*. When Wheat blast disease emerged in Bangladesh in 2016 (Ceresini et al., 2018), resistant wheat varieties were non-existent in the country, as well as neighboring India. Yet, a resistant variety was released within 2 years because of a resistance gene from *Aegilops* that was previously introgressed and ready for use (Cruz et al., 2016; Velu et al., 2018a; Mahmud, 2019).

In this paper, I will first review some difficulties relating to the use of *Aegilops* species (Supplementary Figure S1). Then, I will provide information on the contribution of *Aegilops* to wheat breeding in terms of identified genes in *Aegilops*, as well as some recent information on how *Aegilops* has contributed to the crisis prevention of Ug99 stem rust and wheat blast disease, which may change perspectives of *Aegilops* species as important sources for wheat breeding.

## Hybridization Barriers Between Wheat and *Aegilops* Species and Crossability Genes

To utilize the genetic resources in the *Aegilops* genus, it is necessary to first produce hybrids between wheat and *Aegilops* species. Wheat can be either a female or male parent of the F_1_s, depending on species and specific cross combinations.

**FIGURE 1:**
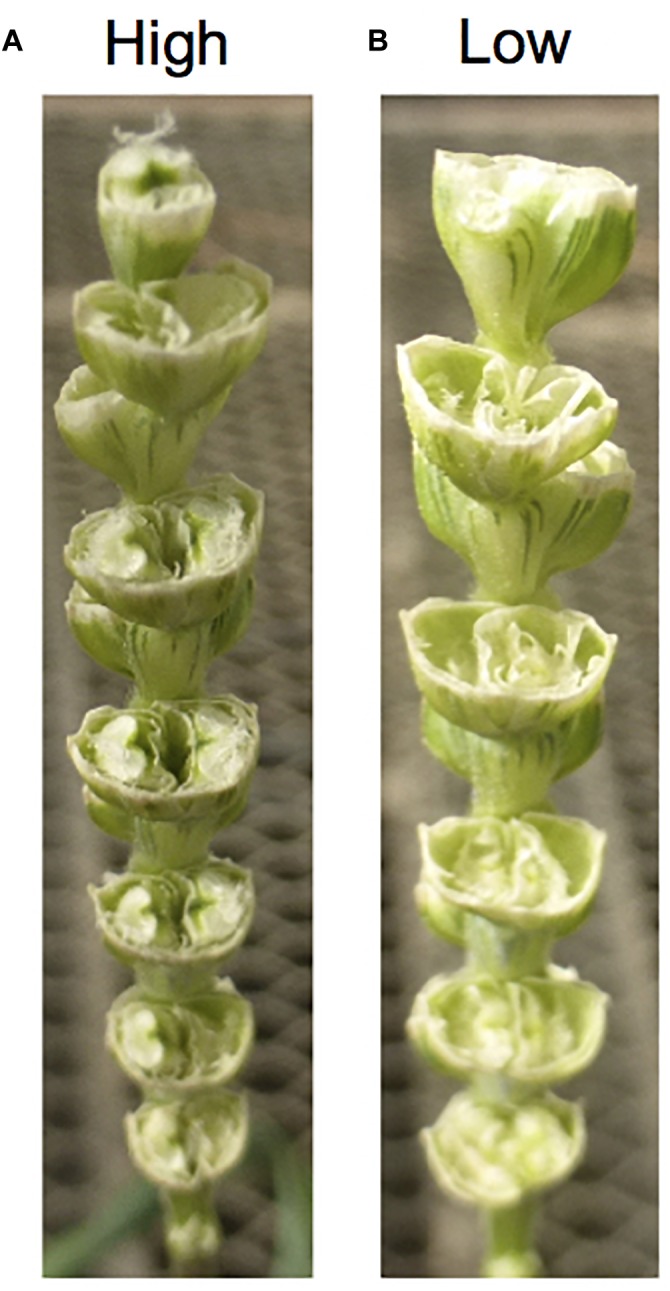
Effects of crossability on seed setting. **(A)** A high crossable durum line. **(B)** A low crossable durum line. The durum spikes show seed setting 2 weeks after pollination with *Ae. tasuchii*. The high crossable line **(A)** sets six grains, while the low crossable line **(B)** sets zero grains.

In wheat × *Aegilops* crosses, crossability genes on the wheat side have been highlighted for their significant role on the success rate of obtaining F_1_ hybrids with *Aegilops* species (Figure 1). This is a key point considering it is very difficult to produce F_1_s using low crossable wheat parents. While East Asian wheat landraces generally have higher crossability success rates with *Aegilops* and other alien species (e.g., rye), European ones have lower rates of success (Zeven, 1987), presumably because European wheat has had greater chances to cross-pollinate with rye histroically. Even though crossability is a QTL trait and controlled by several genes (Alfares et al., 2009), two dominant genes *Kr1* (5BL) and *Kr2* (5AL) were two major genes (Lein, 1943) affecting pollen tube growth (Riley and Chapman, 1967). These two genes have effects across different species including *Hordeum* and *Aegilops* (Snape et al., 1979; Koba and Shimada, 1993). *Kr1* has a stronger effect than *Kr2*, and dominant alleles (*Kr1* and *Kr2*) have inhibition effects. Plants with *Kr1Kr2* show less than 10% crossability, *Kr1kr2* showed between 10 and 25% crossability, *kr1Kr*2 between 25 and 50% and plants with the *kr1kr2* genotype more than 50% crossability (Lein, 1943). Additionally, crossability genes were also reported as *Kr3* on 5D (homoeologous of *Kr1* and *Kr2*) and *Kr4* on 1A (Krolow, 1970; Zheng et al., 1992). More recently, *SKr* on 5BS was reported to have a stronger crossable effect than *Kr1* (Tixier et al., 1998; Alfares et al., 2009; Mishina et al., 2009).

If it is too difficult to produce F_1_ hybrids in wheat × certain *Aegilops* species (wheat as females), pollination in the opposite cross direction (*Aegilops* as females) may be more successful. Dale et al. (2017) reported 0% seed setting in bread wheat × *Ae. tauschii* crosses (probably due to a crossability problem of the bread wheat parents), while it was 30% in *Ae. tauschii* × bread wheat. The seed-setting rate with *Aegilops* as female parents is variable across these species. Yuan et al. (2017) reported the rate was about 0.2% in *Ae. speltoides* × bread wheat, 2–9% in *Ae. cylindrica*, 12–15% in *Ae. ovata* and 22–47% in *Ae. tauschii*. It must be cautioned that the seed setting does not always mean success in obtaining F_1_ plants.

## Endosperm and Embryo Development Deficiency and Embryo Rescue

Gill et al. (1981) observed endosperm abortion and embryo lethality or semi-lethality and seedling death in crosses between *Ae. tauschii* and three diploid *Triticum* species. While the reaction types were different in each three *Triticum* species, the same thing is common in *Ae. tauschii* × bread wheat crosses. Even though the initial seed-setting rate was a 47% (Yuan et al., 2017), the seedling formation rate dropped to 1%. Sehgal et al. (2011) reported that an average of 35% initial embryo formation ended in an average of 7% F_1_ plants.

**FIGURE 2:**
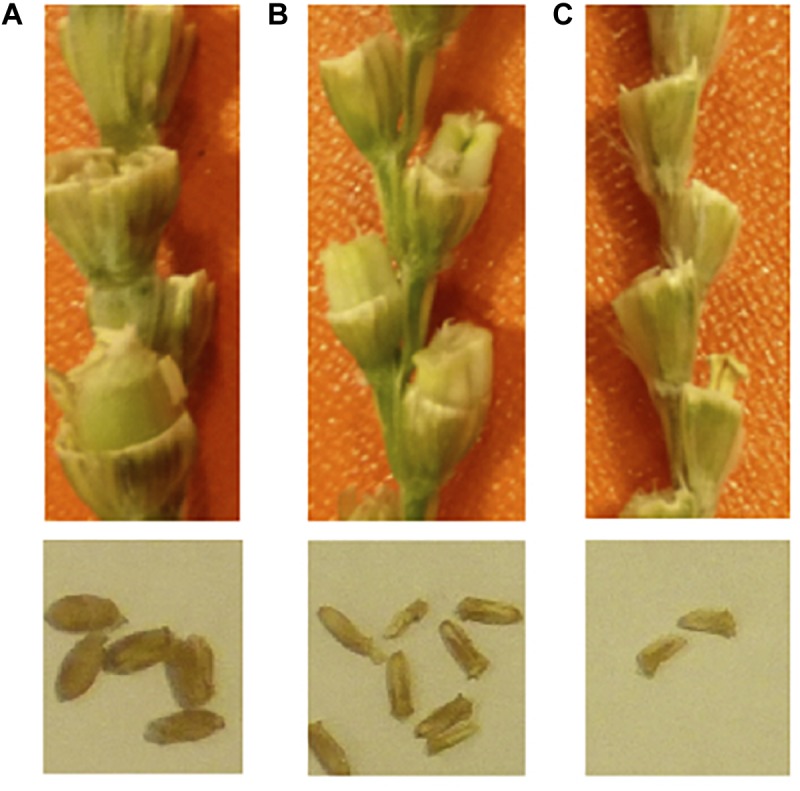
Endosperm development deficiency in F_1_ grains. **(A)** normal selfed-seeds of durum wheat, **(B)** durum wheat cv. LANGDON × *Ae. tauschii*; **(C)** durum wheat cv. CIRNO × *Ae. tauschii*. The seed sizes are smaller in durum × *Ae. tauschii*. The panel **(B)** has some amount of endosperm, and the seeds can germinate. The panel **(C)** has no endosperm, and the seeds will not germinate. Embryo rescue is necessary on the right.

The degree of endosperm development deficiency is cross-combination specific. However, high polyploidy *Aegilops* tend to set endosperm more when crossed with wheat, while diploid *Aegilops* species set less (data not shown). To overcome endosperm abortion, embryo rescue is necessary to recover hybrid seedlings. In this procedure, embryos are dissected from developing grains and transferred to an agar medium with nutrients such as sugar and salts for proper development (Miller et al., 1987). While some wheat lines such as Langdon (durum wheat) or various East Asian landrace lines tend to develop enough endosperm for the embryo to form seeds (Koba and Shimada, 1993), the amount of endosperm sometimes will be lower than normal “wheat × wheat crosses” (Figure 2). It is possible to skip embryo rescue if using these lines. The genetic background of forming unreduced gametes in wheat is not known yet.

## Overcoming Sterility of F_1_S and Unreduced Gametes

The genome of F_1_s between wheat and *Aegilops* in haploids causes sterility until doubling the chromosome numbers. One option is to conduct direct backcrossing of F_1_s with wheat as a pollen donor. Even though the rate of seed set is extremely low, it is possible to obtain BC_1_ plants (Cox et al., 1990; Fritz et al., 1995; Zemetra et al., 1998; Olson et al., 2013). The alternative is through chemical treatments such as colchicine (Blakeslee and Avery, 1937; Tang and Loo, 1940; Bennett and Smith, 1979) and N_2_O gas (Hansen et al., 1988). Some wheat lines such as Langdon produce unreduced gametes, which is a gamete with a 2n nucleus resulting from abnormal meiosis (Fukuda and Sakamoto, 1992; Cai et al., 2010) that leads to spontaneous amphidiploid formation. The formation of unreduced gametes have been reported in durum × *Ae. tauschii*, *Ae. speltoides*, *Ae. longissima*, *Ae. umbellulata*, *Ae. comosa*, *Ae. ovata*, ( = *Ae. geniculata*), *Ae. ventricosa*, *Ae. crassa* and *Ae. triuncialis* (Xu and Dong, 1992; Matsuoka and Nasuda, 2004; Tiwari et al., 2008; Fakhri et al., 2016). The rate of formation is different among *Aegilops* species and prevented by the presence of a shared homologous subgenomes (Fakhri et al., 2016). Additionally, it depends on the genotype of the *Aegilops* parents (Matsuoka and Nasuda, 2004; Fakhri et al., 2016).

## Hybrid Necrosis/Weakness Abnormality

**FIGURE 3:**
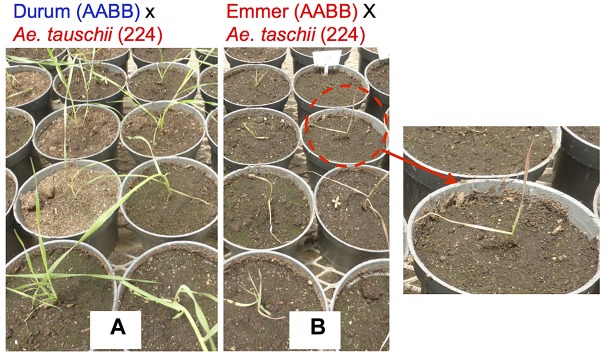
Hybrid necrosis observed in tetraploid wheat × *Ae. tauschii*. **(A)** F_1_ from *Triticum turgidum* ssp. durum cv. CIRNO C 2008 × *Ae. tauschii* WX (224); all plants of this group are growing normally. **(B)** F_1_ from *Triticum* turgidum ssp. dicoccum (PI 233433) × *Ae. tauschii* WX (224); all plants are showing a necrosis symptom of reddish leaf color.

Hybrid necrosis, chlorosis and bushy plant formation is very common in “normal” wheat × wheat cross (Hermsen, 1963; Hermsen and Waninge, 1972; Pukhalskiy et al., 2000; Chu et al., 2006). The *Ne1-Ne2* necrosis system is the best known hybrid necrosis system in wheat, which is caused when two complementary genes of *Ne1* (5BL) and *Ne2* (2BS) are found in the same plant (Tsunewaki, 1960; Nishikawa et al., 1974; Chu et al., 2006). However, this phenomenon is more frequent and complex in wheat × *Aegilops* crosses. Necrosis in *T. turgidum* × *Ae. tauschii* was first reported in the 1960s (Nishikawa, 1960, 1962a,b; Figure 3). Mizuno et al. (2010) did further analysis using a set of synthetic wheat lines that had one common durum wheat parent “Langdon” and different *Ae. tauschii* accessions. They found four different types of hybrid abnormality and responsible genes *Net1* (7DS), *Net2* (2DS), and Hybrid chlorosis1 (*Hch1*; 7DS) in *Ae. tauschii* (Mizuno et al., 2010, 2011; Nakano et al., 2015). The mode of action of these genes should be complementary with genes on the durum side, because the hybrid abnormalities take place only when *Ae. tauschii* is crossed with durum wheat. Hypersensitive response-like reactions were observed for *Net1* necrosis, indicating that it is a kind of disease response reaction (Jeuken et al., 2009; Mizuno et al., 2010). Okada et al. (2017) also reported growth abortion and grass-clump dwarf phenotype in durum × *Ae. umbellulata*. They also showed a repressed expression of the shoot meristem maintenance-related and cell cycle-related genes in the plants with the grass-clump dwarf phenotype. To avoid a problem with hybrid seedling death, Dhaliwal et al. (1986) reported the suppression of *Ne1-Ne2* necrosis at high temperatures. The author also confirmed that incubation at 28°C suppressed necrosis in F_1_s between emmer × *Ae. tauschii* (Supplementary Figure S2). However, the high temperature causes pollen sterility.

## Gametocidal Genes

A group of gametocidal genes (*Gc*), sometimes considered as selfish genes, is another type of obstacle in which the genes cause chromosome breakages in gametes without *Gc* (Endo and Tsunewaki, 1975; Maan, 1975; Endo and Katayama, 1978). This happens when a plant becomes heterozygous in *Gc* —half of the gametes will have *Gc* and the other half will have no *Gc*. Gametes without *Gc* show reduce fitness, which is to the advantage of gametes with *Gc* for the transmission to the next generation (For review, see Tsujimoto, 2005; Endo, 2007; Niranjana, 2017). *Gc* genes have been identified in accessions of certain species that have C, S, S^l^, or M genomes and mostly confined to three different homoeologous groups: 2, 3, and 4 (Endo, 2007). The identified genes include chromosome 3C of *Ae. markgrafii* ( = *Ae. caudata*) and *Ae. triuncialis* (Endo and Tsunewaki, 1975), 2C of *Ae cylindrica* (Endo, 1979), 2S^l^ and 4S^l^ of *Ae. longissima*, 2S^sh^ and 4S^sh^ of *Ae. sharonensis* (Maan, 1975; Endo, 1985), 2S and 6S of *Ae. speltoides* (Tsujimoto and Tsunewaki, 1984, 1988; Kota and Dvorak, 1988) and 4M of *Ae. geniculata* (Kynast et al., 2000). The effects of *Gc* genes are variable; some cause lethality to gametes, while others are mild, allowing incorporation of the gamete into progenies. King et al. (2018) reported the presence of a 2S chromosome segment in all of the developed wheat-*Ae. speltoides* introgression lines due to the gametocidal effect. When researchers use these species, it is better to keep in mind that extra difficulties may arise from *Gc* genes. The suppression of *Gc* genes was reported in Norin 26, which inhibits *Ae. triuncialis Gc3-C1* action and is designated as *Igc1* (Tsujimoto and Tsunewaki, 1985). The presence of additional suppressor genes can also be predicted because the effect of a *Gc* gene is different in various wheat backgrounds. The *Gc* of chromosome 3C is usually lethal but when found in “Chinese Spring” background, it is mild. In addition, Friebe et al. (2003) produced a mutant of the *Ae. sharonensis Gc2* gene (designated as *Gc2mut*) which has a suppression effect on *Gc2*, which will be useful to reduce problems of *Gc* genes in wheat breeding scheme.

## The Use of *Ae. tauschii* For Wheat Breeding

*Aegilops tauschii* is the easiest species in this genus to utilize in wheat breeding, because there is little to no inhibition to meiotic chromosome pairing with the D genome chromosomes of bread wheat. According to several sources, bread wheat originated about 10,000 years ago (Wang et al., 2013; Matsuoka and Takumi, 2017), which is relatively recent and not long enough for genomic differentiation. Furthermore, *Ae. tauschii* contrasts with diploid A genome ancestors. Luo et al. (2000) reported about a 1/6 recombination-rate reduction between *Triticum monococcum* 5A and bread wheat 5A chromosomes when compared to the recombination rate between two *T. monococcum* 5A homologous chromosomes. Even though the A genome of bread wheat and that of the diploid ancestor can form perfect bivalents during meiosis in the F_1_s of AAB (Gill et al., 1988), there are likely to be significant differences in base sequences and chromosome structures (such as inversion, translocations, deletion/duplications, or heterochromatin structures) after the tetraploid wheat formation – i.e., 100,000–500,000 years ago (Huang et al., 2002).

The spontaneous formation of bread wheat in nature was a rare event during which only a very limited number of *Ae. tauschii* plants were involved, based on molecular data and field observations (Dvorak et al., 1998; Matsuoka, 2011; Wang et al., 2013). The genetic diversity of *Ae. tauschii* is far greater in comparison to bread wheat’s D genome diversity (Dvorak et al., 1998; Wang et al., 2013). Matsuoka et al. (2013) proposed sub-dividing *Ae. tauschii* into three groups, TauL1, L2, and L3, and found that bread wheat is close to TauL2 but distinct from TuL1. Even though it is not obvious as in the case of *T. monococcum*, crosses of bread wheat with *Ae. tauschii* accessions of TauL1 may show a reduction in chromosome recombination rates of the A-genome chromosomes.

**FIGURE 4:**
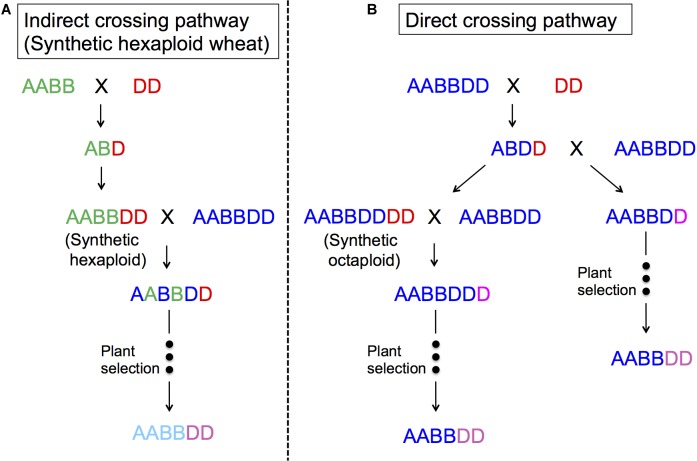
The use of *Ae. tauschii* in wheat breeding. **(A)** indirect crossing pathway (synthetic hexaploid pathway) through durum × *Ae. tauschii* crosses; **(B)** direct crossing pathway through bread wheat × *Ae. tauschii* crosses.

Figure 4 represents two ways to utilize *Ae. tauschii* in wheat breeding, either through direct crossing or indirect crossing (synthetic wheat). With indirect crossing, tetraploid wheat (AABB) will be crossed with *Ae. tauschii* (DD) to produce an F_1_ (ABD), and subsequently this F_1_ will have its chromosome number doubled naturally or artificially to produce so-called synthetic wheat (AABBDD). Synthetic wheat can then be used in wheat breeding by crossing with bread wheat. Synthetic wheat lines were first developed in the United States and Japan in 1940s (Kihara, 1944, 1946; McFadden and Sears, 1944). During the next few decades, a number of synthetic wheat lines were developed by various groups (Kihara and Lilienfeld, 1949; Tanaka, 1961; Dyck and Kerber, 1970; Kerber and Dyck, 1979; Hatchett et al., 1981; Chèvre et al., 1989; Valkoun et al., 1990; Lange and Jochemsen, 1992; Lutz et al., 1994; Wang et al., 2006). Later in the 1980s, CIMMYT started a large-scale production of synthetic wheat, developing more than 1,000 lines (Das et al., 2016; Li et al., 2018). Matsuoka et al. (2007) also reported another set of “Langdon” synthetic wheat lines, and Zeng et al. (2016) produced synthetic wheat using local Chinese land races that were more adaptable to China.

In the direct crossing pathway, *Ae. tauschii* (DD) is crossed with bread wheat (AABBDD) to make an F_1_ (ABDD). These F_1_s are then backcrossed with the same bread wheat (AABBDD) to generate BC_1_, where the plant selection process begins. Gill and Raupp (1987) and Cox et al. (1992) reported this method as successful for transferring Hessian fly and rust resistance. The merit of this method is that it will only change the D genome, making it easy to perform some analyses, as well as directly improving the “best” line without contribution from durum wheat. One of disadvantage of this method may be sterility of the F_1_ plants even as females, and as such, it is necessary to backcross a large number of spikes to have enough BC_1_ seeds to introgress the whole genome (Cox et al., 1990; Fritz et al., 1995; Olson et al., 2013). It is important to note that the seed setting rates in F_1_ plants also depend on *Ae. tauschii* accessions (Matsuoka and Takumi, 2017).

**FIGURE 5:**
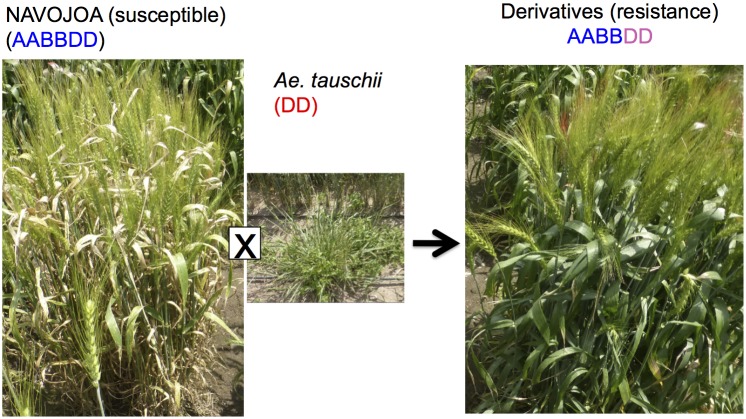
Improvement of Septoria disease resistance through synthetic octaploid wheat. The derivative shows resistance while wheat parent line “NAVOJOA” is susceptible.

Octaploid synthetic wheat is another way to utilize *Ae. tauschii* in wheat breeding, in which an F_1_ (ABDD) from bread wheat (AABBDD) × *Ae. tauschii* (DD) has its chromosome number doubled to produce an octaploid synthetic wheat (AABBDDDD) (Chèvre et al., 1989). Sehgal et al. (2011) and Zhang D. et al. (2018) reported the production of five and one AABBDDDD lines, respectively. CIMMYT has also produced a few hundred octaploid synthetic wheat lines (Supplementary Figure S3). This research resulted in the successful transfer of a dormancy QTL (Dale et al., 2017) and Septoria tritici Blotch resistance (Figure 5).

Table 2 summarizes the resistance genes identified and/or transferred into wheat, including leaf rust, stem rust, stripe rust, powdery mildew, and Hessian fly resistance. It is difficult to identify genes related to abiotic stress (drought and heat) and yield potential, as these traits are not obvious by sight. However, synthetically derived lines have shown up to a 30% yield increase under rain-fed conditions, and a 45% yield increase under drought condition over their wheat parents (Narasimhamoorthy et al., 2006; Dreccer et al., 2007; Trethowan and Mujeeb-Kazi, 2008; Li et al., 2014) and better performance under heat (Iehisa and Takumi, 2012; Jafarzadeh et al., 2016). The percentage of synthetic derivative lines (SDLs) in the Semiarid Wheat Yield Trial reached 52% in 2010, with a five-year average (2010–2015) of 35%. At least, 62 wheat varieties were released using CIMMYT synthetic wheat in their pedigree around the world since 2003 (Li et al., 2018). Elbashir et al. (2017) reported that synthetic derivative lines are promissive for improving heat tolerance in the analysis of multiple synthetic derivative (MSD) lines that cover the whole diversity of *Ae. tauschii*.

**Table 2:** Identified or transferred biotic resistance genes of *Ae. tauschii* into wheat.

Disease/pest	Gene (s)	Method	References
Leaf rust	*Lr21*	SHW	Dyck and Kerber, 1970
	*Lr22a*	SHW	Rowland and Kerber, 1974
	*Lr32*	SHW	Kerber, 1987
	*Lr41*	Direct	Cox et al., 1994
	*Lr42*	Direct	Cox et al., 1994
Stem rust	*Sr33*	SHW	Dyck and Kerber, 1970
	*Sr45*	SHW	Marais et al., 1998
	*Sr46*	SHW	Yu et al., 2015
Stripe rust	*Yr28*	SHW	Singh et al., 2000
Powdery mildew	*Pm2a*	SHW	Lutz et al., 1995
	*Pm19*	SHW	Lutz et al., 1995
	*Pm34*	Direct	Miranda et al., 2006
	*Pm35*	Direct	Miranda et al., 2007
	*Pm58*	Direct	Wiersma et al., 2017
Septoria tritici	*Stb5*	SHW	Arraiano et al., 2001
	*Stb16*	SHW	Ghaffary et al., 2012
Septoria nodorum	*Snb3*	SHW	McIntosh et al., 2008
Tan spot	*Tsr3* ( = *tsn3*)	SHW	Tadesse et al., 2006
Cyst nematode	*Cre3*	SHW	Eastwood et al., 1991
	*Cre4*	SHW	Eastwood et al., 1994
Root knot nematode	*Rkn1*	SHW	Kaloshian et al., 1990
Hessian fly	*H13*	SHW	Gill et al., 1987
	*H22*	Direct (D)	Raupp et al., 1993
	*H23*	Direct (D)	Raupp et al., 1993
	*H24*	Direct (D)	Raupp et al., 1993
	*H26*	SHW	Cox and Hatchett, 1994
Greenbug	*Gb3*	SHW	Hollenhorst and Joppa, 1983
	*Gb4*	SHW	Martin et al., 1982
	*Gb7*	SHW	Weng et al., 2005
	(*Gba*, *Gbb*, *Gbc*,	SHW	Zhu et al., 2005
	*Gbd*, *Gbx2*,	SHW	”
	*Gbx1*, *Gbz*)^∗^	Direct	”
Russian wheat aphid	*Dn3*	SHW (D)	Nkongolo et al., 1991
Wheat curl mite	*Cmc1*	Direct (D)	Thomas and Conner, 1986
	*Cmc4*	Direct	Malik et al., 2003
Soil-Borne Cereal Mosaic Virus	*SBWMV* ( = allelic of S*bm1*?)	Direct	Hall et al., 2009


*^∗^It may be allelic of Gb3. SHW, synthetic hexaploid wheat; Direct, direct crossing; (D), *Ae. tauschii* as female parents. The species name of disease/pest are following: Leaf rust (*Puccinia recondita*), Stem rust (*Puccinia graminis*), Stripe rust (*Puccinia striiformis*), Powdery mildew (*Erysiphe graminis*), Septoria tritici (*Mycosphaerella graminicola*), Septoria nodorum (*Mycosphaerella graminicola*), Tan spot (*Pyrenophora tritici-repentis*), Cyst nematode (*Heterodera avenae*), Root knot nematode (*Meloidogyne* spp.), Hessian fly (*Mayetiola destructor*), Green bug (*Schizaphis graminum*), Russian wheat aphid (*Diuraphis noxia*), Wheat curl mite (*Eriophyes tulipae*), Root knot nematode (*Meloidogyne* spp.) and Soil-Borne Cereal Mosaic Virus.*

## The Use of Tetraploid/Hexaploid *Aegilops* Species With a D Genome

**FIGURE 6:**
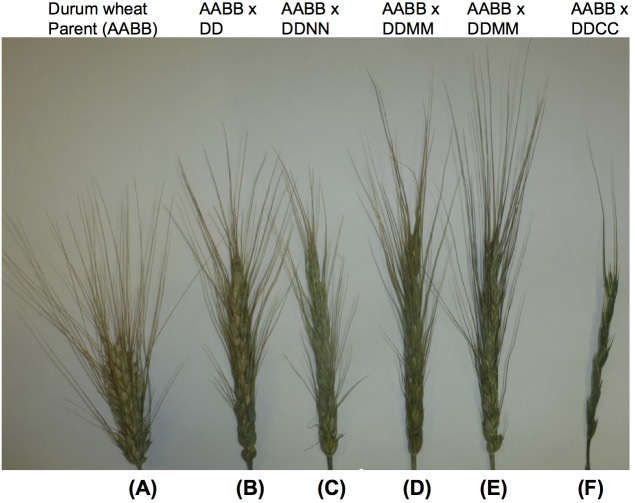
Amphidiploid lines derived from between durum and diploid or tetraploid *Aegilops* with a D genome. **(A)** durum wheat cv. CIRNO C 2008, (AABB); **(B)** durum cv. CIRNO C 2008 × *Ae. tauchii* (895), (AABBDD); **(C)** durum cv. CIRNO C 2008 × *Ae. ventricosa* (PI 542385), (AABBDDNN); **(D)** durum cv. ACONCHI 89 × *Ae. crassa* (PI 542385), (AABBDDMM); **(E)** durum cv. CIRNO C 2008 × *Ae. crassa* (PI 542385), (AABBDDMM); **(F)** durum cv. CIRNO C 2008 × *Ae. cylindrica* (PI 639294), (AABBDDCC). All amphidiploids were produced and maintained at CIMMYT.

Hybrids between tetraploid *Aegilops* species with the D genome can show meiotic pairing with the D genome chromosomes of bread wheat (Kimber and Zhao, 1983). These species include *Ae. cylindrica*, *Ae. crassa*, and *Ae. ventricosa*, *Ae. juvenalis* and *Ae. vavilovii*. Amphiploids of wheat with *Ae. crassa* have been reported (Jovkova et al., 1977; Xu and Dong, 1992), and a number of amphiploids of *Ae. ventricosa* × durum wheat were produced (Delibes et al., 1987). CIMMYT has also developed 20 amphiploid lines of these species using durum and bread wheat (Figure 6) for bread wheat D genome improvement (Supplementary Figure S4).

The eye spot resistance gene *Pch1* (one of two strong seedling resistance genes) was transferred from an amphiploid (AABBDDNN) between tetraploid wheat (AABB) × *Ae. ventricosa* (DDNN) (Table 2). This amphiploid was crossed with bread wheat (AABBDD) to have a derivative line named “VPM1” (Maia, 1967; Doussinault et al., 1983). It was later determined that the location of the transferred *Pch1* is chromosome 7D (Mena et al., 1992).

## The Use of Other *Aegilops* Species and Chromosome Pairing

For the use of other *Aegilops* species, a reduced chromosome pairing frequency is more problematic. *Ae. cylindrica*, *Ae. crassa*, *Ae. ventricosa*, *Ae. Juvenalis*, and *Ae. vavilovii* are also categorized in this group due to the presence of non-D genomes.

Because of the lack of recombination, it is common to produce so-called alien chromosome addition or substitution lines in which one pair of *Aegilops* (or alien) chromosomes is added to or substituted for a pair of wheat chromosomes, respectively (Supplementary Figure S5). A number of addition lines have been produced from 14 different *Aegilops* species (For a review see; Schneider et al., 2008; Kilian et al., 2011). These addition lines are very useful for analysis and locating useful genes at the chromosomal level. However, addition lines have less breeding value, because they have many negative factors and the presence of an extra alien chromosome disrupts the genetic harmony of a genome. To be more appropriate for breeding, it is necessary to produce introgression lines (small *Aegilops* chromosome segment transfers) or Robertsonian/centromeric translocation lines (Robertson, 1916), in which one of the *Aegilops* chromosome arms is translocated to a wheat chromosome, replacing an arm of that wheat chromosome (Supplementary Figure S5). These translocations can be obtained spontaneously from addition/substitution lines or amphiploids in backcrossing populations, or using wheat monosomic lines (2*n* = 41; with one of the homoeologous chromosomes is missing). All of these can lead to the occurrence of univalent chromosomes during meiosis. Then, the meiotic spindle fiber will attach to the both sides of univalent chromosomes, which then causes chromosome breakages through the centromeric regions at high frequency. The broken chromosome arms are sticky and may fuse to other broken chromosomes to produce centromeric translocations (Supplementary Figure S6).

Homoeologous meiotic pairing between chromosomes of wheat and *Aegilops* species is inhibited mostly by the *Ph1* gene (5BL) (Okamoto, 1957; Riley and Chapman, 1958; Sears and Okamoto, 1958; Riley, 1960). Therefore, the meiotic barrier can be overcome by suppressing of *Ph1* activity. Sears (1977) produced the *ph1b* mutant in which the *Ph1* locus is missing and this is the most widely used *Ph1* gene mutant in wheat breeding. Another gene which affects homoeologous chromosome pairing was identified as *Ph2* (3D) (Mello-Sampayo, 1971) and has a mild inhibition effect on *Ph1* (Sears, 1982). Additional mutants, *ph1c* (Giorgi, 1983) and *ph2* (Sears, 1982) are also available, even though they have been rarely used in the breeding. It is also known that the presence of *Ph1* suppressors or promotors of homoeologous chromosome paring are present in some accessions of *Aegilops* species: *Ae. speltoides* (Feldman and Mello-Sampayo, 1967; Dover and Riley, 1977; Dvorak et al., 2006), *Ae. longissima* (7), *Ae. mutica* (Dover and Riley, 1972), *Ae. umbellulata* (Riley et al., 1973), *Ae. Peregrina*, and *Ae. kotschyi* (Fernandez-Calvin and Orellana, 1991) and *Ae. geniculata* (Koo et al., 2017). Therefore, the transfer of traits may be easier in accessions that have the suppressive effects. The *Ae. speltoides* genes are considered to be suppressants, because they can promote more meiotic pairing in the presence of the *Ph1* gene (Dover and Riley, 1972). A couple of suppressor genes of *Ae. speltoides* has been transferred into wheat and designated as *PhI* (Chen et al., 1994) and *Su1-Ph1* (7S) and *Su2-Ph1* (3S) (Dvorak et al., 2006; Li et al., 2017). Since these genes are dominant, they can be faster and easier to utilize in breeding. Yet the effects of *PhI* have been shown to be lower than that of *ph1b* (Aghaee-Sarbarzeh et al., 2000).

Sometimes it is difficult to induce homoeologous recombination due to different homoeologous co-linearity between wheat and *Aegilops* chromosomes (Molnár et al., 2013, 2016). It is also known that centromeric and other chromosomal regions may have very low recombination rates, even in wheat × wheat crosses (Saintenac et al., 2009). In these cases, other methodologies become an alternatives. Sears (1956) demonstrated a successful transfer of *Ae. umbellulata Lr9* gene into wheat using irradiation. Yet this is the only success story using irradiation for introgression of *Aegilops* chromatin for wheat breeding until recently. Singh et al. (2016) and Verma et al. (2016) recently reported the production of a translocation by irradiation of *Ae. kotschyi* hybrids. Mild effect *Gc* genes and some chemicals can also induce random translocations, much like irradiation. Even though it is not for breeding purposes, the *Gc* system has been used for producing translocations of wheat-rye and wheat-barley (Joshi et al., 2013; Li et al., 2013; Ishihara et al., 2014).

## Useful Genes of *Aegilops* Transferred to Wheat

Through the use of the various techniques described above, a number of genes have been transferred from *Aegilops* (including *Ae. tauschii*) to wheat (Tables 3, 4). In terms of total number, leaf rust resistance genes are the most numerous (20), followed by powdery mildew (15), and green bug (12). Since more than 75 resistance gene loci have been identified and permanently designated as resistance genes by 2018 (Ponce-Molina et al., 2018). *Aegilops* provided more than 20% of them. For powdery mildew, 54 resistance loci were found by 2018 (Tang et al., 2018), and *Aegilops* contributed about 20%. For Cereal Cyst Nematodes (CCN) resistance genes, a total of 12 genes have been identified, including *Cre1-8*, *CreR*, *CreV*, *CreX*, *CreY* (Ali et al., 2019). Of them, two (*Cre1* and *Cre8*) are indigenous to the wheat genepool. The others are from *Ae. tasuchii* (*Cre3* and *Cre4*), *Ae. ventricosa* (Zhuk.) (*Cre2*, *Cre5*, and *Cre6*), *Ae. triuncialis* L. (*Cre7*); *Ae. peregrina* (*CreX* and *CreY*), *Secale cereale* (*CreR*) and *Dasypyrum villosum* (*CreV*) (Zhang et al., 2016), showing that two thirds of them are from *Aegilops*. In terms of actual species of origin, *Ae. tauschii* has provided the most number of genes, followed by *Ae. speltoides* and then *Ae. ventricosa*. It is worth noting that most of the disease resistances from *Ae. ventricosa* are provided by a single 2NS-2AS translocation, including *Lr37*, *Sr38*, *Yr17*, *Cre5*, *Rkn3* (Bariana and McIntosh, 1993, 1994; Jahier et al., 1996, 2001; Helguera et al., 2003; Tanguy et al., 2005; Williamson et al., 2013); this translocation has originated from VPM1 (Maia, 1967) that also has *Pch1* resistance on 7D (Mena et al., 1992).

**Table 3:** Identified or transferred biotic resistance genes in *Aegilops* (other than from *Ae. tauschii*) into wheat.

Disease/pest		Genome	Gene	References
Eyespot	*Ae. ventricosa*	DN	*Pch1*	Doussinault et al., 1983
		(recombination between two D genomes)		
Leaf rust	*Ae. umbellulata*	U	*Lr9*	Sears, 1956
			*Lr76*	Bansal et al., 2017
	*Ae. speltoides*	S	*Lr28*	McIntosh et al., 1982
			*Lr35*	Kerber and Dyck, 1990
			*Lr36*	Dvorak and Knott, 1990
			*Lr37*	Bariana and McIntosh, 1993
			*Lr47*	Helguera et al., 2000
			*Lr51*	Helguera et al., 2005
			*Lr66*	Marais et al., 2009a
	*Ae. kotschyi*	US	*Lr54*	Marais et al., 2005
	*Ae. sharonensis*	S^sh^	*Lr56*	Marais et al., 2010
	*Ae. geniculata*	UM	*Lr57*	Kuraparthy et al., 2007
	*Ae. triuncialis*	UC	*Lr58*	Kuraparthy et al., 2011
	*Ae. peregrina*	US	*Lr59*	Marais et al., 2008
	*Ae. neglecta*	UM	*Lr62*	Marais et al., 2009b
Stem rust	*Ae. speltoides*	S	*Sr32*	McIntosh, 1988
			*Sr39*	Kerber and Dyck, 1990
			*Sr47*	Klindworth et al., 2012
	*Ae. comosa*	M	*Sr34*	McIntosh et al., 1982
	*Ae. ventricosa*	DN	*Sr38*	Bariana and McIntosh, 1993
	*Ae. searsii*	S^s^	*Sr51*	Liu et al., 2011a
	*Ae. geniculata*	UM	*Sr53*	Liu et al., 2011b
Stripe rust	*Ae. comosa*	M	*Yr8*	Riley et al., 1968
	*Ae. ventricosa*	DM	*Yr17*	Bariana and McIntosh, 1993
	*Ae. kotschyi*	US	*Yr37*	Marais et al., 2005
	*Ae. sharonensis*	S^sh^	*Yr38*	Marais et al., 2010
	*Ae. geniculata*	UM	*Yr40*	Kuraparthy et al., 2007
	*Ae. neglecta*	UM	*Yr42*	Marais et al., 2009b
	*Ae. umbellulata*	U	*Yr70*	Bansal et al., 2017
Powdery mildew	*Ae. speltoides*	S	*Pm1d*	Hsam et al., 1998
			*Pm12*	Jia et al., 1996
			*Pm32*	Hsam et al., 2003
			*Pm53*	Petersen et al., 2015
	*Ae. longissima*	S^l^	*Pm13*	Donini et al., 1995
	*Ae. geniculata*	UM	*Pm29*	Zeller et al., 2002
	*Ae. umbellulata*	U	*Pm57*	Liu et al., 2017
Cyst nematode	*Ae. ventricosa*	DN	*Cre2*	Delibes et al., 1993
			*Cre5*	Jahier et al., 1996
			*Cre6*	Ogbonnaya et al., 2001
	*Ae. triuncialis*	UC	*Cre7*	Romero et al., 1998
	*Ae. peregrina*	US	(*CreX*)	Barloy et al., 2007
			(*CreY*)	Barloy et al., 2007
Root knot nematode	*Ae. peregrina*	US	*Rkn2*	Yu et al., 1990
	*Ae. ventricosa*	DN	*Rkn3*	Williamson et al., 2013
Hessian fly	*Ae. ventricosa*	DN	*H27*	Delibes et al., 1997
	*Ae. triuncialis*	UC	*H30*	Martin-Sanchez et al., 2003
Green bug	*Ae. speltoides*	S	*Gb5*	Friebe et al., 1991


*The species name of disease/pest are following: Eyespot (*Tapesia yallundae*), Leaf rust (*Puccinia recondita*), Stem rust (*Puccinia graminis*), Stripe rust (*Puccinia striiformis*), Powdery mildew (*Erysiphe graminis*), Cyst nematode (*Heterodera avenae*), Root knot nematode (*Meloidogyne* spp.), Root knot nematode (*Meloidogyne* spp.), Hessian fly (*Mayetiola destructor*) and Green bug (*Schizaphis graminum*).*

**Table 4:** List of resistance gene against stem rust Ug99 race.

Origin of Sr genes	Effective Sr genes				
*Triticum aestivum*	*Sr9h*	*Sr15*^∗1^	*Sr28*	*Sr42*	+ 2 temporal^∗2^
(Partial; APR^∗a^)	*Sr55*	*Sr56*	*Sr57*	*Sr58*	
*Triticum dicoccum*	*Sr2*	*Sr13*			
*Triticum timopheevi*	*Sr36*	*Sr37*			
*Triticum araraticum*	*Sr40*				
*Triticum monococcum*	*Sr21*	*Sr22*	*Sr35*	*Sr60*	+ 2 temporal^∗3^
*Aegilops tauschii*	*Sr33*	*Sr45*	*Sr46*		+ 3 temporal^∗4^
*Aegilops speltoides*	*Sr32*	*Sr39*			
*Ae. sharonensis*					+ 3 temporal^∗5^
*Aegilops searsii*	*Sr51*				
*Aegilops triuncialis*	*Sr47*				
*Aegilops geniculata*	*Sr53*				
*Aegilops umbellulata*					+ 1 temporal^∗6^
*Thinopyron ponticum*	*Sr24*	*Sr25*	*Sr26*	*Sr43*	
*Thinopyrum intermedium*	*Sr44*				
*Secale cereale*	*Sr27*	*Sr50*	*Sr59*		+ 1 temporal^∗7^
*Dasypyrum villosum*	*Sr52*				


*The table was constructed according to Kielsmeier-Cook et al. (2015) and Randhawa et al. (2018) with updates by the author. ^∗*a*^Partial resistance genes; APR, adult plant resistance gene; ^∗*1*^Data from multiple research groups are not consistent (Singh et al., 2015); ^∗*2*^*3SrND643* (Basnet et al., 2015), SrTmp, SrCad (cosegregating with Sr42; Hiebert et al., 2016); ^∗*3*^*Sr60* (Chen et al., 2018), SrTm4 (Briggs et al., 2015), SrTm5 (Chen et al., 2018); ^∗*4*^*SrTA10171*, *SrTA10187*, *SrTA1662*; ^∗*5*^*SrSha7* (Singh et al., 2015); *Sr-1644-1S^*sh*^* and *Sr-1644-5S^*sh*^* (Yu et al., 2017); ^∗*6*^2U chromosome (Edae et al., 2016); ^∗*7*^*Sr59* (Rahmatov et al., 2016), *Sr1RSAmigo.**

Recently, *Aegilops* has gathered more attention for improving micro-nutrient content (such as Fe and Zn) in wheat grains. Zn deficiency affects 17.3% of the world’s population across Asia and Africa, leading to the deaths of more than 400,000 children each year (Cakmak, 2007; Black et al., 2013; Velu et al., 2018b). Micro-nutrient rich wheat, i.e., bio-fortified wheat, can improve the lives of these people. It is difficult to find high Zn and Fe content germplasm in the wheat genepool (Cakmak et al., 2010), even though some *Aegilops* species show three to four-fold higher Zn and Fe grain content, including *Ae. longissima* (S^l^), *Ae. kotschyi* (US), *Ae. peregrina* (US), *Ae. cylindrica* (CD), *Ae. ventricosa* (DN), *Ae. geniculata* (UM) (Rawat et al., 2009). Amphiploid durum- *Ae. longissima* and partial amphiploids of wheat – *Ae. kotschyi* show two to three times higher levels of Zn and Fe grain content than the parental wheat line (Tiwari et al., 2008, 2010). Rawat et al. (2011) further reported Zn grain content three times higher in wheat- *Ae. kotschyi* addition/substitution lines than the wheat parent.

In addition to the benefit for wheat breeding mentioned above, it is also important to highlight that *Aegilops* introgression lines have a level of diversity and unique traits that wheat lacks. Even though these are of no immediate benefit at this moment, their value could be seen in the future, as exemplified by two recent global wheat production threats.

## A Story of *Aegilops* Translocations on Stem Rust UG99 Race

A serious threat to global wheat production is the emergence of stem rust Ug99 race, which was recognized in Uganda in 1999 (Pretorius et al., 2000). This disease had the potential to develop into a global catastrophe, as more than 70% of wheat varieties around the world did not have resistance against Ug99 in the early 2000s (Singh et al., 2015). Many wheat breeders and pathologists, who had thought stem rust was no longer a problem, were caught unprepared and were then spurred to search for new resistant sources. The researchers realized that while the bread and durum wheat gene pools do not have many resistant sources, resistance is available outside the genepool from ancestral and alien species including many in *Aegilops* (Table 4, based on Yu et al., 2014; Kielsmeier-Cook et al., 2015; Randhawa et al., 2018 with updates by the author). This has also promoted various studies to identify new stem rust resistance genes, which led the identification of *Sr46* (*Ae. tauschii*; Yu et al., 2015), *Sr47* (*Ae. triuncialis*; Klindworth et al., 2012), *Sr51* (*Ae. searsii*; Liu et al., 2011a), *Sr53* (*Ae. geniculate*; Liu et al., 2011b) and three additional genes in *Ae. tauschii* (Rouse et al., 2011), three genes in *Ae. sharonensis* (Singh et al., 2015; Yu et al., 2017) and one gene in *Ae. umbellulata* (Edae et al., 2016). In addition, it has been reported that 81% of *Ae. longissima* (out of 394 accessions), 94% of *Ae. neglecta* (189 out of 202 accessions tested), 88% of *Ae. cylindrica* (DDCC) and *Ae. peregrina* (SSUU) were Ug99 resistant (Huang S. et al., 2018; Olivera et al., 2018).

Even though introgression lines of two Ug99 resistance genes (*Sr32* and *Sr39*) from *Ae. speltoides* were available, they were not used in wheat breeding program due to the presence of large *Ae. speltoides* segments and associated negative factors on agronomy (Friebe et al., 1996). Fortunately, researchers in Australia and the United States started preparing for the possible appearance of dangerous new stem rust pathogen races back in the early 1990s and the reports of Ug99 just confirmed their expectations. Based on that work, shortened introgressions of chromosome 2S segments with *Sr32* and *Sr39* were already developed using the *ph1b* mutant and have been quickly distributed around the world (Mago et al., 2009, 2013; Niu et al., 2011).

It is notable that it has eight resistance genes (+ three temporary assigned genes) in the bread wheat gene pool are effective to Ug99, but four of them (*Sr55*, *Sr56*, *Sr57*, and *Sr58*) are partial or adult plant resistance genes (APR), so it is necessary to combine them with other genes to exert a higher level of resistance (Gustafson and Shaner, 1982; McIntosh et al., 1995, 1998, 2012).

## A Story of the 2NS Translocation in Relation to Wheat Blast Disease

Wheat blast caused by *Pyricularia oryzae* (*Magnaporthe oryzae*) is an emerging disease that was first recognized in Brazil in the 1980s (Igarashi et al., 1986). The pathogen gained an ability to infect the new host plant wheat through a mutation of an avirulence gene (Inoue et al., 2017). Since then, it has been a serious obstacle for wheat production in central and south Brazil, south-east Paraguay and eastern Argentina, affecting 300 million ha of wheat fields and reducing the yield of infected areas 100–10% (Kohli et al., 2011; Perello et al., 2015; Duveiller et al., 2016). The disease jumped to Bangladesh in 2016 and spread to 15,000 ha (Malaker et al., 2016). Because of this serious threat to the wheat production of South Asia, quick remedial action was required to prevent a devastating epiphytotic (Mottaleb et al., 2018). Eight different resistance genes against wheat blast (*Rmg1-8*) have been reported, and only two of them (*Rmg7* and *Rmg8*) are effective in the field in Bangladesh (Anh et al., 2017). Since *Rmg7* and *Rmg8* recognize the same avirulence gene peptide of the pathogen, both resistance genes are functionally equivalent to a single gene for resistance (Anh et al., 2017). Despite of lacking resistance sources, a new resistance wheat variety, “BARI com” was released in Bangladesh within 2 years in 2018. This happened because of the existence of the 2NS-2AS translocation (Cruz et al., 2016; Velu et al., 2018a; Mahmud, 2019). This translocation has been utilized in wheat breeding programs because of rust resistances (Juliana et al., 2017), but it also happens to have a strong wheat blast resistance. If 2NS-2AS had not have been produced, the wheat blast issue would have been a much more serious problem in the last few years. Another amazing finding with the 2NS-2AS translocation is that nearly 90% of advanced lines of the CIMMYT bread wheat program have this translocation (Juliana et al., 2017; Philomin Juliana, Personal communication). As in the case of the T1BL.1RS translocation that dominated wheat cultivars for decades, a beneficial translocation can have a huge impact on wheat breeding and production.

## The Use of *Aegilops* in the Genomic Age for Breeding and Pre-Breeding

During the last several decades, cytogenetic methods not only have been essential tools for screening and understanding the nature of translocations and alien introgressions from a number of progenies, but also possess the major constraint in handling large numbers of samples. But new cytogenetic FISH/GISH technology using oligo probes expands the capacity, proving a valuable tool in cytogenetics (Du et al., 2017; Huang X. et al., 2018). More importantly, recent progress in high through-put genotyping technology and availability of molecular methods makes it possible to detect alien segments very easily, which has been promoting the production of alien segment introgressions. Niu et al. (2011) screened about 1,000 plants and found 40 smaller alien recombinants of *Ae. speltoides* 2S chromosome using the *ph1b* mutant. The development of translocations which cover a whole genome have been demonstrated in *Ambylopyrum mutica* ( = *Ae. mutica*) (King et al., 2017) and *Ae. speltoides* (King et al., 2018). The number of estimated introgression segments obtained are about 200 of *Am. mutica* (King et al., 2017), and a map of about 600 cM was made with 540 plants in the case of *Ae. speltoides* (King et al., 2018), which allowed the construction of linkage maps even using wheat- *Aegilops* introgression lines and the Axiom 35K SNP array that was constructed on a wheat sequence based Axiom 820K SNP array by optimizing for finding polymorphism between wheat and *Aegilops* species. An increased number of whole genome linkage or physical maps in *Aegilops* species have been available (Table 5). A 4-gigabase physical map based on BAC clones of *Ae. tauschii* led the construction of a 10 K *Ae. tauschii* Infinium SNP array (Luo et al., 2013). Moreover, the draft sequence of *Ae. tauschii* has been recently reported (Luo et al., 2017), and a TILLING population of *Ae. tauschii* was reported (Rawat et al., 2018). It will be possible to have additional physical maps and draft sequences in another *Aegilops* species in near future that will facilitate their use in wheat breeding and gene identifications.

**Table 5:** List of whole or semi-whole genome genetic or physical maps in *Aegilops* species.

Species name	Type of markers	Type of populations	References
*Aegilops tauschii*	RFLP^∗1^	F2 of *Ae. tauschii*	Gill et al., 1991
	RFLP; SSR^∗2^	F2 and F3 of *Ae. tauschii*	Boyko et al., 2002
	SSR	F2 of *Ae. tauschii*	Okamoto et al., 2013
	10K SNP array of *Ae. tauschii*	F2 of *Ae. tauschii*	Luo et al., 2013
	EST^∗3^; SSR; RJM^∗4^	RH^∗10^ of synthetic wheat	Kumar et al., 2012
	DArT^∗5^; SSR	RH of *Ae. tauschii*	Kumar et al., 2015
	SSR	F2 of *Ae. tauschii*	Nishijima et al., 2018
*Aegilops speltoides*	RFLP	F2 of *Ae. speltoides*	Dvorak et al., 2006
	Axiom 35K SNP array	Wheat/*Ae. speltoides* introgressions	King et al., 2018
*Aegilops longissima*	RFLP	F2 of *Ae. longissima*; CS/*Ae. longissima* addition	Zhang et al., 2001
	SSR	RIL^∗11^ of *Ae. longissima*	Sheng et al., 2012
	RNA-seq^∗6^	CS/*Ae. longissima* addition	Wang et al., 2018
*Aegilops sharonensis*	DArT; SSR	F2 of *Ae. sharonensis*	Olivera et al., 2013
	OPA^∗7^	RIL and F2 of *Ae. sharonensis*	Yu et al., 2017
*Aegilops umbellulata*	RFLP	CS/*Ae. umbellulata* addition	Zhang et al., 1998
	GBS^∗8^	F2 of *Ae. umbellulata*	Edae et al., 2016
	GBS	F2 of *Ae. umbellulata*	Edae et al., 2017
*Aegilops caudata*	SSR	CS/*Ae. caudata* addition	Niu et al., 2018
*Aegilops comosa*	PAUG^∗9^	CS/*Ae. comosa* addition	Liu et al., 2019
*Amblyopyrum mutica*	Axiom 35K SNP array	Wheat/*Am. mutica* introgressions	King et al., 2017


*^∗*1*^RFLP, restriction fragment length polymorphism; ^∗*2*^SSR, simple sequence repeat; ^∗*3*^EST, expression sequence tag; ^∗*4*^RJM, repeat DNA junction marker; ^∗*5*^DArT, diversity arrays technology; ^∗*6*^RNA-seq, RNA sequencing; ^∗*7*^OPA, oligo pool assay; ^∗*8*^GBS, genotyping-by-sequence; ^∗*9*^PAUG, PCR-based landmark unique gene; ^∗*10*^RH, radiation hybrid; ^∗*11*^RIL, recombinant inbred line.*

Yet the biggest limitation and challenge for the use of *Aegilops* is still reduced recombination rates between wheat and *Aegilops* chromosomes that is sometimes prohibitive in producing an *Aegilops* introgression segment. The new technologies such as MutChromSeq (mutant chromosome sequencing), MutRenSeq (Mutagenesis Resistance gene enrichment and sequencing) and AgRenSeq (Association Genetics R gene enrichment Sequencing) may provide an alternative to overcome gene identification obstacles. These techniques allow a rapid isolation of mutated genes with mutagenesis by sequencing sorted chromosomes (MutChromSeq) or enriching target gene families by exome capture (MutRenSeq) or a rapid isolation of natural variants by enriching target gene family (AgRenSeq) and sequencing for resistance gene homologs. Steuernagel et al. (2016) reported the cloning of *Sr22* and *Sr45* from bread wheat using MutRenSeq, Sánchez-Martín et al. (2016) reported the cloning of *Pm2* using MutChromSeq, and, Arora et al. (2019) demonstrated the discovering and cloning of *Sr33*, *Sr45*, *Sr46*, and *SrTA1662* from a panel of about 200 *Ae. tauschii* accessions using AgRenSeq. Development of new methodologies which can compensate the reduced recombination rate may overcome the biggest constrains of the use of *Aegilops*. Alternatively, we may be able to find new variations or genes to increase the recombination rate from *Aegilops* like *PhI* genes (Chen et al., 1994; Dvorak et al., 2006; Li et al., 2017).

As we can see from the stories of Ug99 and wheat blast, *Aegilops* species are important not only for pre-breeding but also for a proactive main-stream breeding. It is still necessary to induce a certain level of recombination between wheat and alien chromosomes for the use of *Aegilops*, but the new technologies are opening up a new era of *Aegilops* for wheat breeding.

## Data Availability

All datasets generated for this study are included in the manuscript and/or the Supplementary Files.

## Author Contributions

The author confirms being the sole contributor of this work and has approved it for publication.

## Conflict of Interest Statement

The author declares that the research was conducted in the absence of any commercial or financial relationships that could be construed as a potential conflict of interest.

## Supplementary Material

Figure S1 Various constrains in obtaining F_1_/amphiploids between wheat and *Aegilops* species. In this case, the cross is between bread wheat (AABBDD) and *Ae. speltoides* (SS). Measures corresponds to the constraints of the left.

Figure S2 Suppression of hybrid necrosis in F1s between emmer x *Ae. tauschii* by incubation at 28°C. **(A)**: incubation at 28°C; **(B)**: incubation at 22°C.

Figure S3 Synthetic octaploid and hexaploid wheat lines. **(A)** bread wheat cv. BORLAUG 100 × *Ae. tauchii* (WX 700), (AABBDDDD); **(B)** bread wheat cv. BORLAUG 100 × *Ae. tauchii* (WX 1195), (AABBDDDD); **(C)**: bread wheat cv. BORLAUG 100 × *Ae. tauchii* (KU 2096), (AABBDDDD); **(D)**: bread wheat cv. BORLAUG 100 × *Ae. tauchii* (KU 2811), (AABBDDDD); **(E)**: durum cv. ACONCHI 89 × *Ae. tauchii* (KU 2811), (AABBDD); **(F)**: durum cv. CIRNO C 2008 × *Ae. tauchii* (KU 2811), (AABBDD). DD, *Ae. tauschii*. All amphiploids were produced and maintained at CIMMYT.

Figure S4 The use of the D genome in tetraploid *Aegilops* species.

Figure S5 The use of *Aegilops* species (except *Ae. tauschii*) for wheat breeding. Introgression lines can be produced from any part of F_1_ haploid, amphiploid, addition/substitution lines, and centromeric translocation line.

Figure S6 The mechanism of forming Robertsonian (centromeric) translocation. During meiosis, spindle fibers will attach to the both side of univalent chromosomes, which leads chromosome breakage at the centromeric region at high frequency (the right one). Broken chromosomes may fuse with other broken chromosome arm, forming centromeric translocation. The 3A, 3B, and 3U are 3A, 3B, and 3U chromosomes. S, short arm; L, long arm.

## Abbreviations

BC_1_: backcrossed generation 1
CIMMYT: International Maize and Wheat Improvement Center
SHW: synthetic hexaploid wheat
