# Supplementary figures and images for: An Update of Recent Use of Aegilops Species in Wheat Breeding
Source: Front Plant Sci. 2019 May 9;10:585. doi: 10.3389/fpls.2019.00585 (PMC6521781; doi:10.3389/fpls.2019.00585)

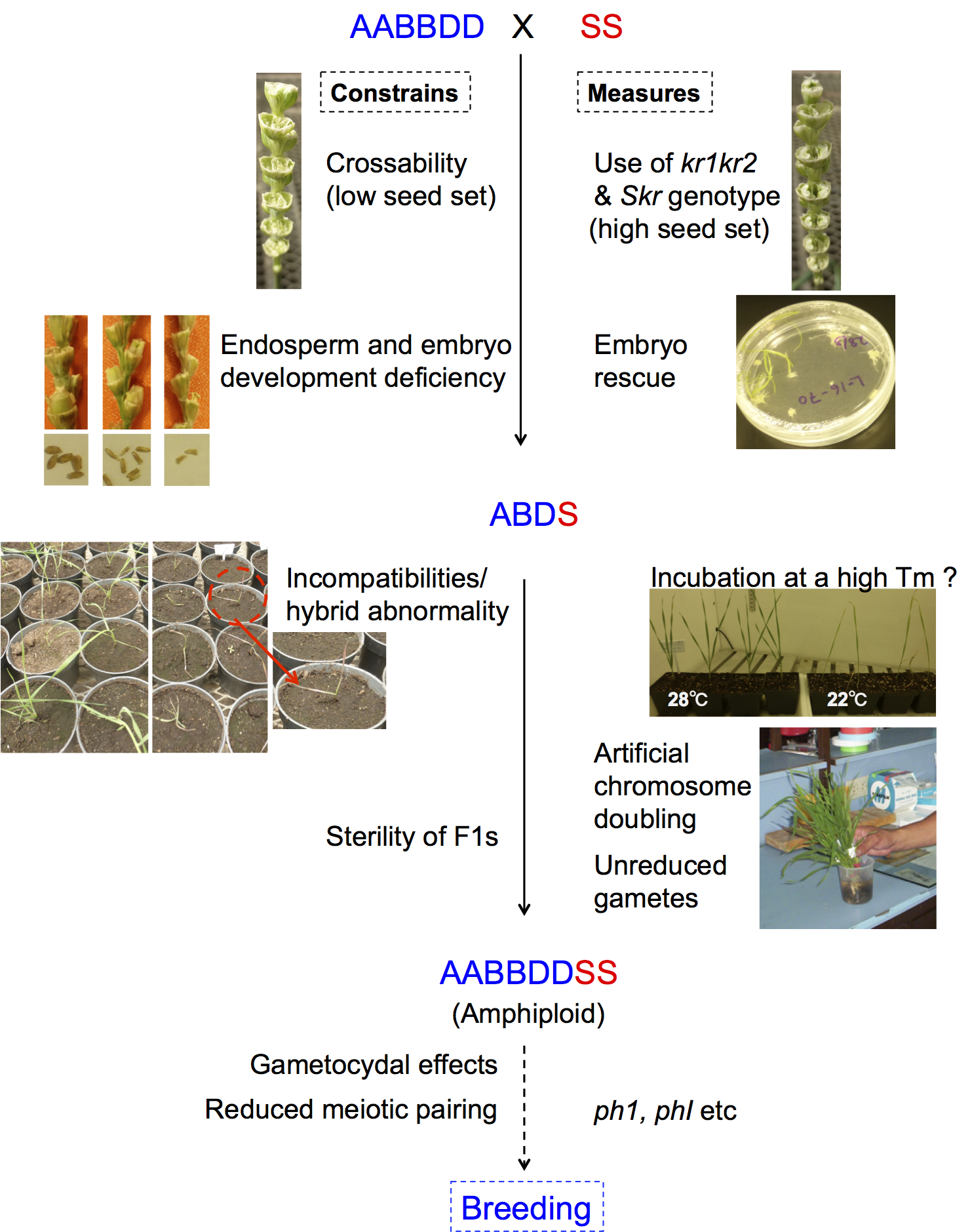

Supplement: Figure S1 — Various constrains in obtaining F1/amphiploids between wheat and Aegilops species. In this case, the cross is between bread wheat (AABBDD) and Ae. speltoides (SS). Measures corresponds to the constraints of the left. [file Image_1.JPEG]

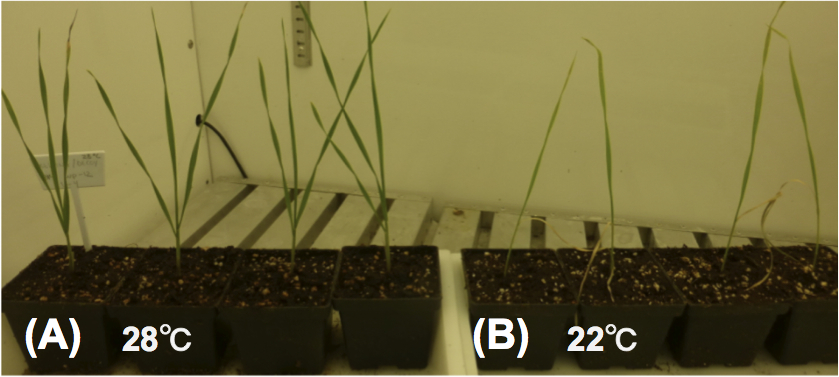

Supplement: Figure S2 — Suppression of hybrid necrosis in F1s between emmer x Ae. tauschii by incubation at 28°C. (A): incubation at 28°C; (B): incubation at 22°C. [file Image_2.JPEG]

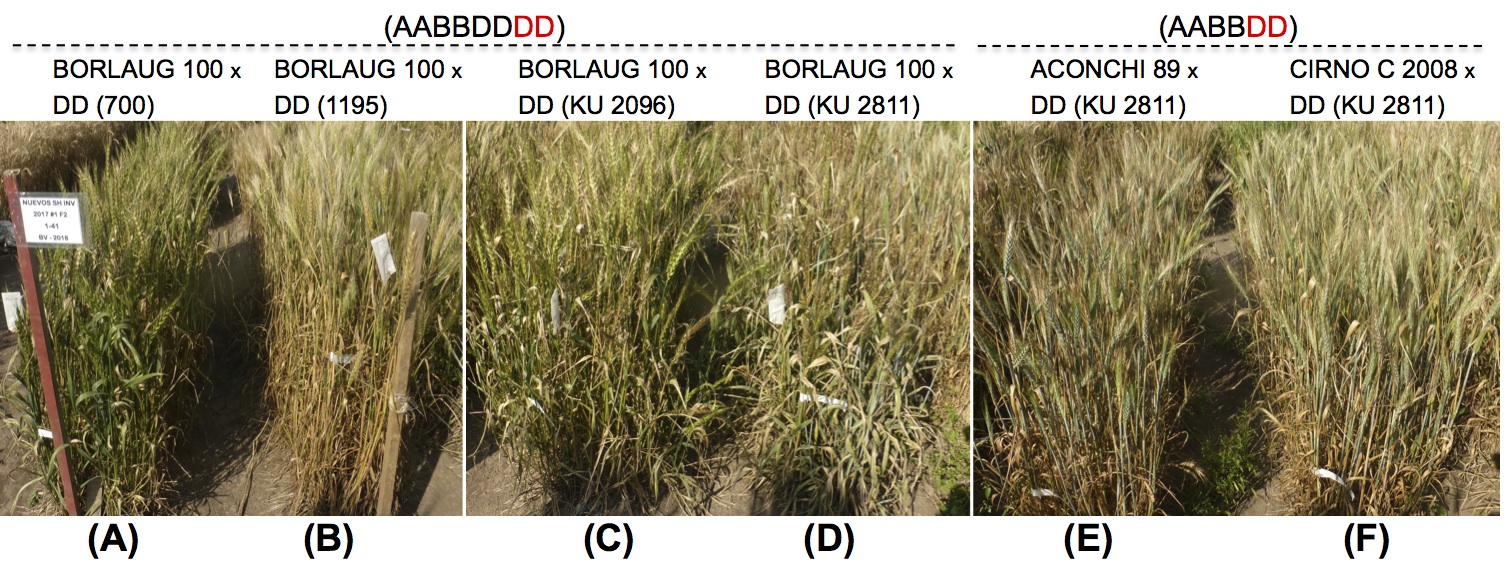

Supplement: Figure S3 — Synthetic octaploid and hexaploid wheat lines. (A) bread wheat cv. BORLAUG 100 × Ae. tauchii (WX 700), (AABBDDDD); (B) bread wheat cv. BORLAUG 100 × Ae. tauchii (WX 1195), (AABBDDDD); (C): bread wheat cv. BORLAUG 100 × Ae. tauchii (KU 2096), (AABBDDDD); (D): bread wheat cv. BORLAUG 100 × Ae. tauchii (KU 2811), (AABBDDDD); (E): durum cv. ACONCHI 89 × Ae. tauchii (KU 2811), (AABBDD); (F): durum cv. CIRNO C 2008 × Ae. tauchii (KU 2811), (AABBDD). DD, Ae. tauschii. All amphiploids were produced and maintained at CIMMYT. [file Image_3.jpg]

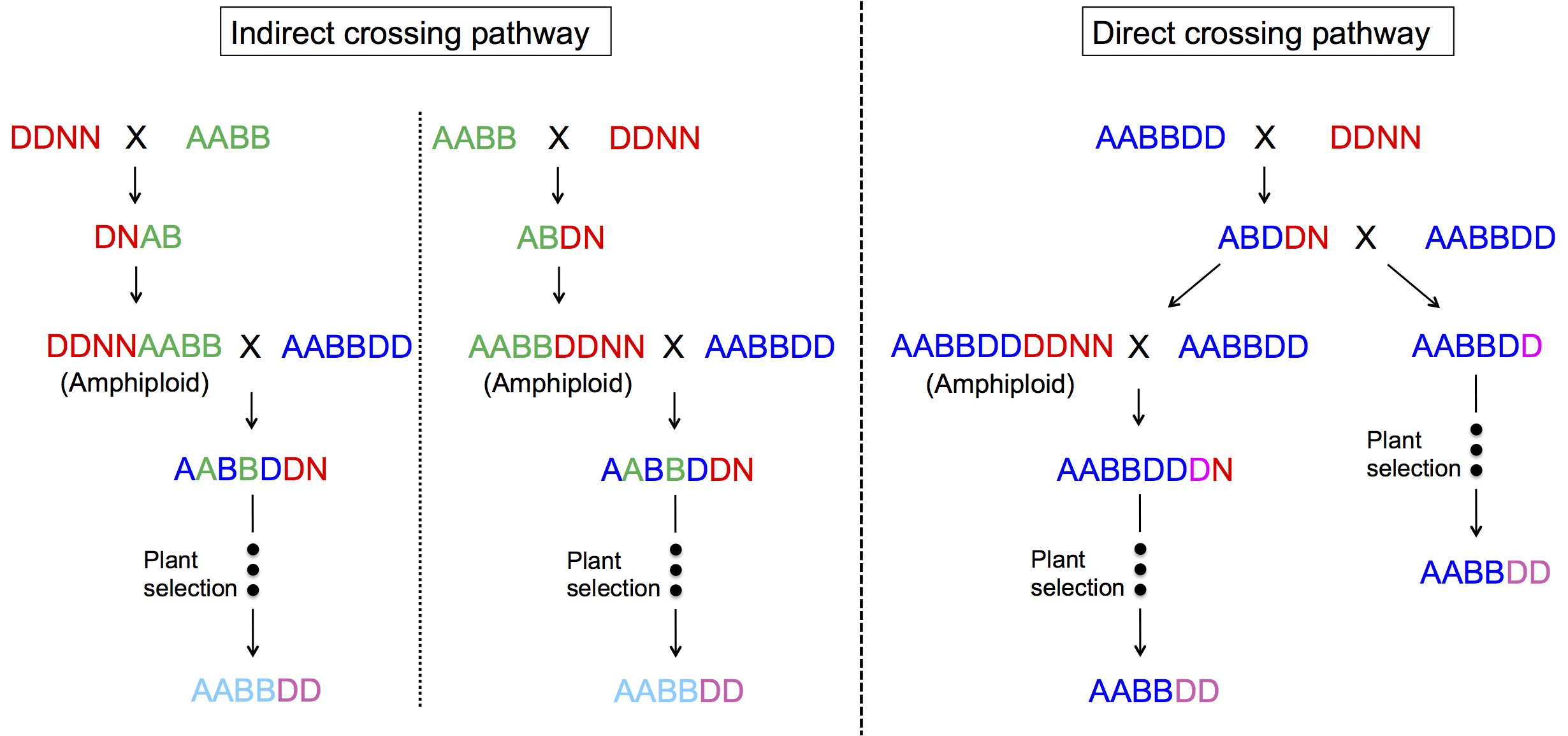

Supplement: Figure S4 — The use of the D genome in tetraploid Aegilops species. [file Image_4.jpg]

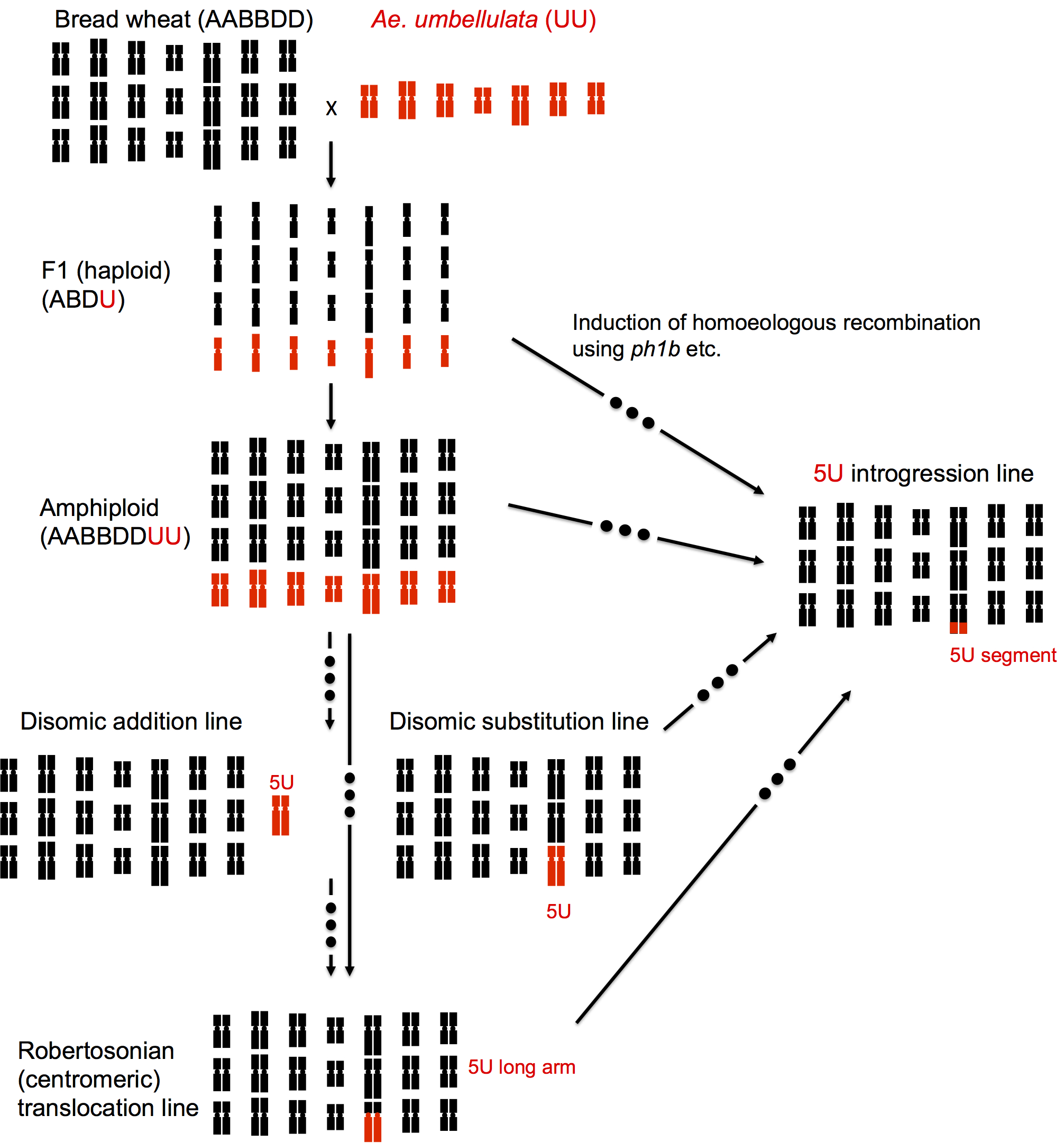

Supplement: Figure S5 — The use of Aegilops species (except Ae. tauschii) for wheat breeding. Introgression lines can be produced from any part of F1 haploid, amphiploid, addition/substitution lines, and centromeric translocation line. [file Image_5.JPEG]

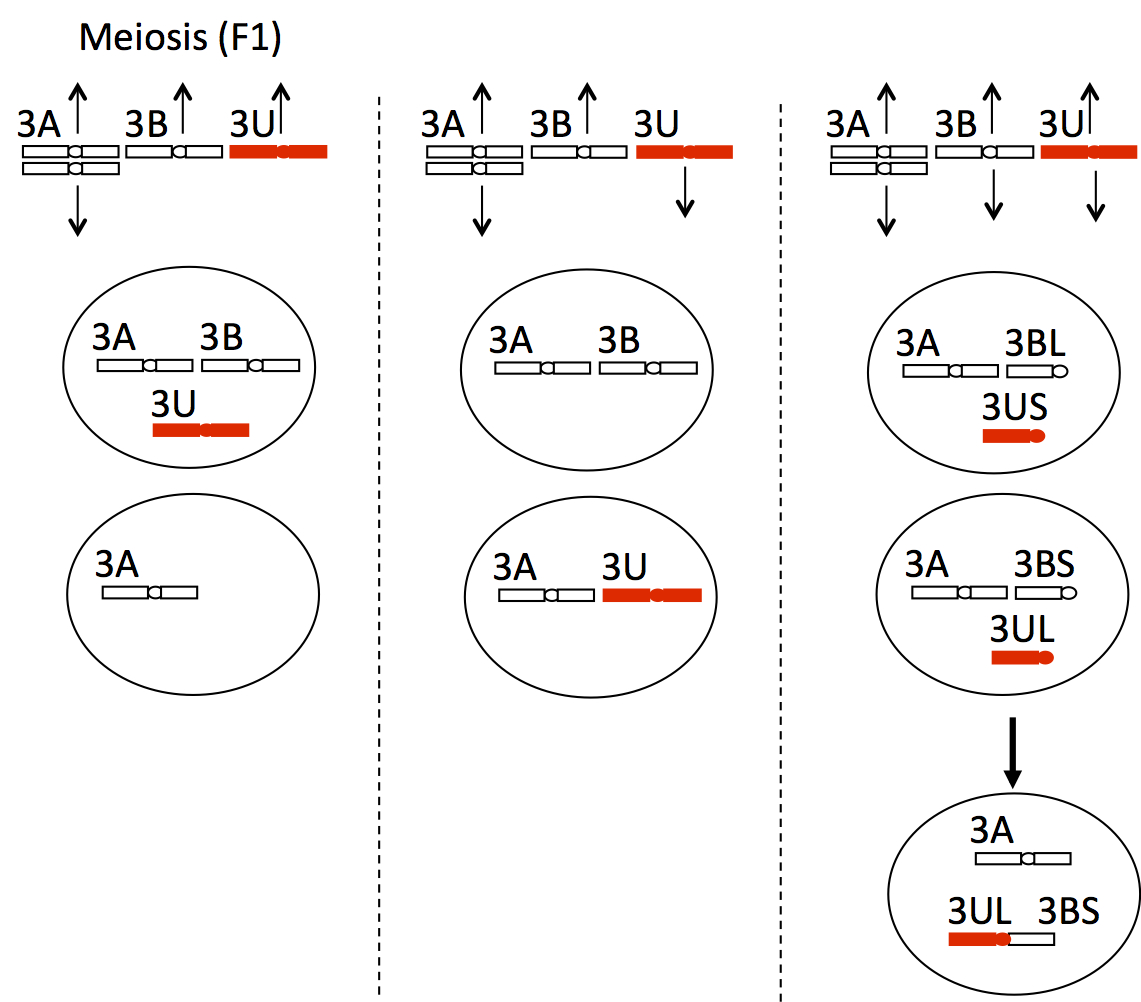

Supplement: Figure S6 — The mechanism of forming Robertsonian (centromeric) translocation. During meiosis, spindle fibers will attach to the both side of univalent chromosomes, which leads chromosome breakage at the centromeric region at high frequency (the right one). Broken chromosomes may fuse with other broken chromosome arm, forming centromeric translocation. The 3A, 3B, and 3U are 3A, 3B, and 3U chromosomes. S, short arm; L, long arm. [file Image_6.JPEG]
